# MADIA - Meteorological variables for agriculture: A dataset for the Italian area

**DOI:** 10.1016/j.dib.2022.108843

**Published:** 2022-12-20

**Authors:** Barbara Parisse, Roberta Alilla, Antonio Gerardo Pepe, Flora De Natale

**Affiliations:** Council for Agricultural Research and Economics–Research Centre for Agriculture and Environment, via della Navicella 4, 00184 Roma, Italy

**Keywords:** ERA5, Reanalysis, Gridded datasets, 10-day, Evapotranspiration, Anomalies, Climate normals, Climate percentiles

## Abstract

The MADIA gridded dataset provides the dekadal series of the main agro-meteorological variables derived from ERA5 hourly surface data, across Italy for the period 1981-2021, and their respective 1981-2010 and 1991-2020 climate normals, also including absolute minimum and maximum and the main quantiles. Temporal and spatial resolutions are 10-day and 0.25 degrees respectively and the dataset is annotated with standard metadata. The dataset was obtained by: (1) estimating the daily time series of minimum, average and maximum air temperature, minimum and maximum air relative humidity, wind speed, surface solar radiation downwards, precipitation and reference evapotranspiration according to the FAO Penman-Monteith method; (2) summarising them to 10-day series as accumulated values for precipitation and evapotranspiration and mean values for the other variables. The MADIA dataset is provided in both NetCDF and csv format. A complementary vector file is provided which reports for every cell the fractions covered of the total area of each administrative unit considered to derive statistics for Italy on the European Nomenclature of Territorial Units for Statistics levels (NUTS 2 and 3). Other potential dataset reuses are the estimation of bioclimatic indices and statistical downscaling of climate scenarios.

**Specifications table**

**Table:** 

Subject	Environmental Science: Climatology
Specific subject area	Agro-meteorological variables and climate normals for crop suitability analysis and climate change assessment in Italy
Type of data	NetCDF embedding metadata (.nc) text table (.csv) vector files (consisting of .shp and associated files)
How the data were acquired	The raw hourly gridded meteorological data were extracted from the Copernicus Climate Change Service ERA5 reanalysis dataset [1] through the Climate Data Store API client (a Python based library) for the following variables: near-surface air temperature, dew point temperature, precipitation, surface solar radiation downwards (shortwave radiation), both wind speed components and geopotential. The layer of the official administrative boundaries was obtained through the repository of the Italian National Institute of Statistics (Istat), updated to the 1st of January 2020 (https://www.istat.it/it/archivio/222527) The data processing was performed by the GIS software QGIS (version 3.22.8, https://download.qgis.org) and Python 3.6 (http://www.python.org)
Data format	Analysed
Description of data collection	The 10-day gridded agro-meteorological data (air temperature and humidity, precipitation, wind speed, shortwave solar radiation downwards, evapotranspiration) was derived from the ERA5 dataset [1], for the area within the coordinates 34.875–48.125 N, 4.875 – 20.125 E which includes the Italian country, in the period 1981-2021. Data spatial resolution is 0.25 degrees. Periodically, new climate data is automatically downloaded and processed and the MADIA dataset [2] is updated. In order to summarise the MADIA variables at the Italian official administrative units (at NUTS 2 and 3 levels) [3], a specific elaboration was performed to derive the weights of each grid cell for every NUTS unit covered by the cell itself, by overlaying the Italian official administrative boundaries [4] with the ERA5 grid in a GIS environment.
Data source location	Institution: Council for Agricultural Research and Economics- Research Centre for Agriculture and Environment City: Rome Country: Italy
Data accessibility	Repository name: Zenodo Data identification number: 10.5281/zenodo.7252361 Direct URL to data: https://doi.org/10.5281/zenodo.7252361

## Value of the Data

• The MADIA gridded dataset [2] makes available additional data resources to support agro-meteorological and hydrological analyses across Italy for the period 1981-2021.
• Agro-meteorological data is crucial for agriculture land suitability analysis, as climate factors play an important role in crop development and growth. This data is also useful for analyses of the agro-meteorological context, at both national and subnational levels, in order to select the more effective measures in support of the policy theme on climate change [5].
• This dataset can be mainly used by the research community, national and regional/local agro-meteorological services, decision makers, federations of farmers.
• MADIA dataset can be used for several aims like e.g. (1) analysing agro-meteorological time series at different temporal scales and NUTS levels; in fact, its granularity of 10-day data can be aggregated to different levels to satisfy various user requirements; (2) perform agro-meteo-climatic analyses by deriving anomalies and bioclimatic indices; (3) statistical downscaling of climate scenarios.

## 1 Objective

The main objective of this dataset is to support researchers and technicians in their agro-meteorological and climatological analyses for Italy, making available pre-processed data derived from ERA5 reanalysis of Climate Data Store Service of Copernicus [1], updated with a sub-annual time step and provided in an easy-to-use text table format. The dataset will allow users who are not experienced in coding or with limited access to computing resources to take advantage of information provided by the ERA5 reanalysis.

## 2 Data Description

The MADIA gridded dataset [2] includes the dekadal series of agro-meteorological variables at 0.25 degrees resolution across Italy for the period 1981-2021 and their respective 1981-2010 and 1991-2020 climatological standard normals.

The list of variables is presented in Table 1.

**Table 1:** The list of variables provided.

Short name	Description
tasmin	mean of daily minimum near-surface air temperature
tasmean	mean of daily average near-surface air temperature
tasmax	mean of daily maximum near-surface air temperature
rhmin	mean of daily minimum near-surface relative air humidity
rhmax	mean of daily maximum near-surface relative air humidity
ws10	mean of daily wind speed
ssrd	mean of daily surface solar radiation downwards (shortwave radiation)
ppn	sum of daily depth of water-equivalent precipitation
pev	sum of daily crop reference evapotranspiration estimated by FAO Penman-Monteith method
zg	geopotential height: average cell height (metres) above the geoid, which corresponds approximately to the elevation
dekad	number of dekad from the beginning of the year
expver	code which identifies temporary data when expver=5
mask	boolean code to identify cells belonging to the Italian country

The data are provided in two formats: an open data cube NetCDF format, embedding metadata, and the CSV formatted data, a form more readily usable by researchers unfamiliar with the data cube format.

Overall, 43 NetCDF files are provided: 41 files contain the annual series of the listed variables, and 2 additional files derive from the computation of the cited climatological standard normals. The data is provided for the entire bounding box.

The same dataset is provided as comma-separated tables in 47 files: 43 data files (41 files contain the annual series of the listed variables, and 2 additional files derive from the computation of the two climatological standard normals) and 3 metadata tables. The latter include 1 file with discovery metadata and 2 files with description metadata for the annual series and the climate normals.

Furthermore, vector data (.shp and associated files) is provided which represents the results of the overlay of the Italian official administrative boundaries [3] with the ERA5 grid in a GIS environment, with the contribution (weight) of each grid cell to the official total area of the NUTS polygons at 2 and 3 levels.

The list of MADIA files is presented in Table 2.

**Table 2:** The MADIA dataset files available on Zenodo.

Content	Folder	Format	File name
annual time series (*YYYY* from 1981 to 2021)	1_nc_data	NetCDF	YYYY_e5_10d.nc
	2_csv_data	text table	YYYY_e5_10d.csv
Climate normals	1_nc_data	NetCDF	19812010_climate_normal_e5_10d_v1.1.nc 19912020_climate_normal_e5_10d_v1.1.nc
	2_csv_data	text table	19812010_climate_normal_e5_10d_v1.1.csv 19912020_climate_normal_e5_10d_v1.1.csv
Discovery and description metadata	3_metadata	text table	e5_10d_discovery_metadata.csv e5_10d_description_metadata.csv e5_10d_climate_normal_description_metadata_v1.1.csv
NUTS2-3 cover fractions	4_shp_data	vector file	ERA5_cells_cover_fraction_4_IT_NUTS.cpg ERA5_cells_cover_fraction_4_IT_NUTS.dbf ERA5_cells_cover_fraction_4_IT_NUTS.prj ERA5_cells_cover_fraction_4_IT_NUTS.qmd ERA5_cells_cover_fraction_4_IT_NUTS.shp ERA5_cells_cover_fraction_4_IT_NUTS.shx

## 3 Experimental Design, Materials and Methods

Among several meteorological gridded datasets covering Italy, some products provided by Copernicus are characterised by a set of variables and a spatial and temporal resolution suitable for agrometeorological analyses. Four datasets are of great interest: the aforementioned ERA5, ERA5 Land (at 0.1 degrees resolution), which has been directly derived from the first one [6], E-OBS (at the same 0.1 degrees resolution) and the most recent CERRA (with a resolution of 5.5 km). Although the latter seems very promising for its horizontal resolution, it should be noticed that, up to now, both the temporal coverage (since 1984) and the update (it is available until June 2021) make it uncompetitive with the other datasets [7]. The best update frequency is offered by ERA5 (5 days latency) and ERA5 Land (2-3 months latency). Although ERA5 Land shows a better spatial resolution, an important limit of this dataset (especially for the Italian peninsula, with approximately 8,000 km of coastline) is that data are not provided for the grid points falling on the sea surface or in the proximity of the coastline [8]. Another point is related to data accuracy: a study carried out in Italy has shown similar or slightly improved performances of ERA5 in comparison to ERA5 Land [9]. Therefore, the disadvantage of managing a larger dataset is not always balanced by an improvement in terms of accuracy. As regards precipitation, it is more difficult to represent its erratic spatial distribution; a study over Central Italy (period: 1951-2019), has shown that ERA5 generally overestimates the annual rainfall, except on the north-central Apennines where it is underestimated [10]. Anyway, with reference to several Italian irrigation districts, a general good agreement was obtained between observed and reanalysis (ERA5 and ERA5 Land) derived agrometeorological variables at both daily and seasonal scales [9]. For these reasons, the dataset presented here is derived from ERA5.

In order to build the MADIA dataset, ERA5 hourly surface data at 0.25 degrees resolution was summarised to the dekadal series of the main agro-meteorological variables and to their 1981-2010 and 1991-2020 climatological standard normals as well as the main climate percentiles.

The raw hourly gridded meteorological data for the bounding box covering Italy in the period 1981-2021 were extracted from the Copernicus Climate Change Service ERA5 reanalysis dataset [1] through the Climate Data Store API client (a Python based library). The following variables were selected: near-surface air temperature, dew point temperature, precipitation, shortwave radiation downwards, both wind speed components and geopotential. Based on this data, the cell's geopotential height and the daily time series of minimum, average and maximum air temperature, minimum and maximum air relative humidity, wind speed, shortwave solar radiation and precipitation were calculated. More specifically, the relative humidity was derived from hourly humidity time series derived in turn from dew point and air temperature. The choice of starting from hourly data allows to reduce bias issues in computing air humidity and in the derived estimate of evapotranspiration [11]. Then, the reference evapotranspiration was obtained using the required daily data according to the FAO Penman-Monteith method [12]. All daily time series were summarised on a 10-day resolution as accumulated values for precipitation and evapotranspiration and mean values for the other variables. The dekadal series were used to compute two climatological standard normals (1981-2010, 1991-2020), as well as the following statistics on the 30-year dekadal values of each variable: absolute minimum and maximum, 5^th^, 10^th^, 50^th^, 90^th^, 95^th^ percentiles.

Moreover, data was annotated with discovery (global attributes, which describe the whole dataset) and description (variable specific attributes) metadata which meet the domain relevant community standards, as required by the FAIR principles [13], including Climate and Forecast (CF) Metadata Convention v1.7 [14], WMO core metadata profile of the ISO 19115 metadata standard [15] and Attribute Convention for Dataset Discovery (ACDD) v1.3 [16].

The MADIA dataset [2] was built by structuring the processed data (with the associated metadata) in NetCDF format, producing a set of annual files and 2 additional ones for the climate normals. Moreover, the same data was organised in text table format (csv files). In this format, each ERA5 grid cell is identified by the latitude/longitude coordinates of its centre.

**Fig. 1:**
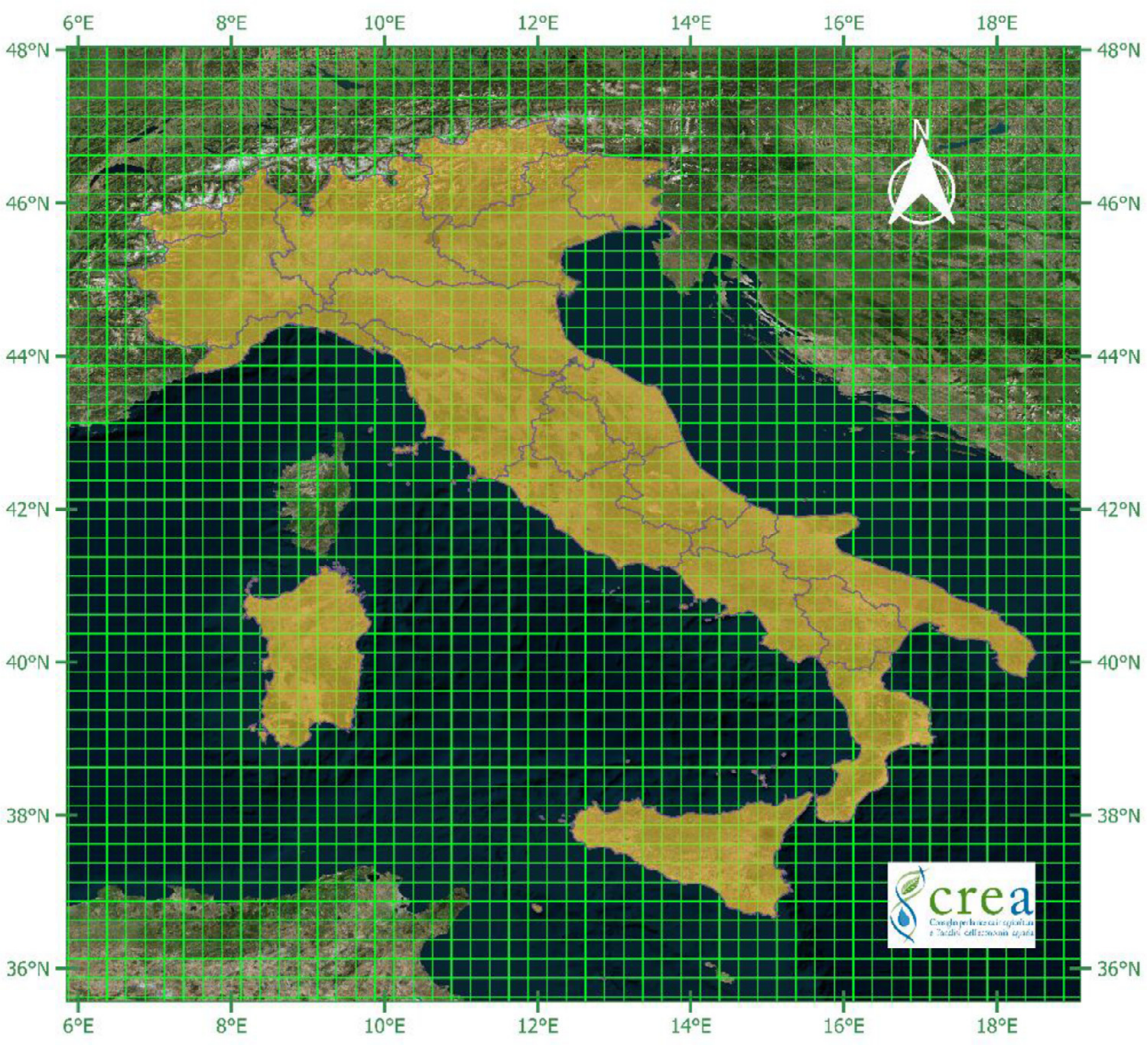
The ERA5 grid overlaid to the Italian NUTS2 units.

For agrometeorological reporting aims, in order to summarise the MADIA variables at the Italian NUTS 2 and 3 levels [3], a specific elaboration was performed to derive the weights of each grid cell in terms of the area fraction (ranging between 0 and 1) of every NUTS unit covered by the cell itself. The Italian official administrative boundaries [4] were overlaid with the ERA5 grid in a GIS environment (Fig. 1) to derive the cell weights. The results are provided directly in a vector file (.shp and associated files, including the. qmd metadata file), which reports the polygons obtained by the overlay representing the cell portions belonging to the different NUTS.

The data processing was performed by the GIS software QGIS (version 3.22.8, https://download.qgis.org) and Python (version 3.6) [17], with the specific MetPy package (version 1.0.1) [18]

The data from which the MADIA dataset is derived is updated every day with the available raw data (near real time) [1] through an automated code to feed the Biophysical Models Application framework (BioMA) [19] with agro-meteorological data as well as to support the requirements from the research community, regional agro-meteorological services and the Italian Ministry of Agricultural, Food and Forestry Policies. The MADIA dataset stored in the Zenodo repository [2] will be periodically aligned with the latest validated ERA5 data available (excluding preliminary data) [20] and will be updated also in relation to the future developments of the Copernicus Climate Data Store.

## Ethics Statements

This dataset did not involve the use of human subjects, animal experiments, nor data collected from social media platforms.

## CRediT Author Statement

**Barbara Parisse:** Conceptualization, Methodology, Software, Data curation, Writing – original draft, Writing – review & editing; **Roberta Alilla:** Methodology, Data curation, Writing – original draft, Writing – review & editing; **Antonio Gerardo Pepe:** Methodology, Software, Data curation, Writing – review & editing; **Flora De Natale:** Methodology, Data curation, Writing – original draft, Writing – review & editing.

## Declaration of Competing Interest

The authors declare that they have no known competing financial interests or personal relationships that could have appeared to influence the work reported in this paper.

## Data Availability

Meteorological variables for Agriculture: a Dataset for the Italian Area (MADIA) (Original data) (Zenodo).
